# Molecular subtype classification of urothelial carcinoma in Lynch syndrome

**DOI:** 10.1002/1878-0261.12325

**Published:** 2018-06-19

**Authors:** Christina Therkildsen, Pontus Eriksson, Mattias Höglund, Mats Jönsson, Gottfrid Sjödahl, Mef Nilbert, Fredrik Liedberg

**Affiliations:** ^1^ The HNPCC register Clinical Research Center Copenhagen University Hospital Hvidovre Denmark; ^2^ Division of Oncology and Pathology Institution of Clinical Sciences Lund University Sweden; ^3^ Division of Urological Research Institution of Translational Medicine Lund University Sweden; ^4^ Department of Urology Skåne University Hospital Malmö Sweden; ^5^ Danish Cancer Society Research Center Copenhagen Denmark

**Keywords:** bladder cancer, lynch syndrome, upper urinary tract urothelial carcinoma, urothelial carcinoma

## Abstract

Lynch syndrome confers an increased risk for urothelial carcinoma (UC). Molecular subtypes may be relevant to prognosis and therapeutic possibilities, but have to date not been defined in Lynch syndrome‐associated urothelial cancer. We aimed to provide a molecular description of Lynch syndrome‐associated UC. Thus, Lynch syndrome‐associated UCs of the upper urinary tract and the urinary bladder were identified in the Danish hereditary nonpolyposis colorectal cancer (HNPCC) register and were transcriptionally and immunohistochemically profiled and further related to data from 307 sporadic urothelial carcinomas. Whole‐genome mRNA expression profiles of 41 tumors and immunohistochemical stainings against FGFR3, KRT5, CCNB1, RB1, and CDKN2A (p16) of 37 tumors from patients with Lynch syndrome were generated. Pathological data, microsatellite instability, anatomic location, and overall survival data were analyzed and compared with sporadic bladder cancer. The 41 Lynch syndrome‐associated UC developed at a mean age of 61 years with 59% women. mRNA expression profiling and immunostaining classified the majority of the Lynch syndrome‐associated UC as urothelial‐like tumors with only 20% being genomically unstable, basal/SCC‐like, or other subtypes. The subtypes were associated with stage, grade, and microsatellite instability. Comparison to larger datasets revealed that Lynch syndrome‐associated UC shares molecular similarities with sporadic UC. In conclusion, transcriptomic and immunohistochemical profiling identifies a predominance of the urothelial‐like molecular subtype in Lynch syndrome and reveals that the molecular subtypes of sporadic bladder cancer are relevant also within this hereditary, mismatch‐repair defective subset.

**Abbreviations**
GU: genomically unstable
HNPCC: hereditary nonpolyposis colorectal cancer
IHC: immunohistochemistry
MMR: mismatch repair
MSI: microsatellite instability
TMA: tissue microarray
UC: urothelial carcinoma
Uro: urothelial‐like
UTUC: upper urinary tract urothelial carcinoma

## 1 Introduction

Heredity in upper urinary tract urothelial carcinoma particularly relates to the Lynch syndrome, which is caused by germline mutations in the mismatch repair (MMR) genes *MLH1*,* MSH2*,* MSH6,* or *PMS2*. The highest risks apply to colorectal cancer (50–80% lifetime risk) and endometrial cancer (40–60% lifetime risk), but efficient surveillance and prophylactic risk reduction procedures have led to improved survival rates and prolonged life expectancy. This implies a longer time at‐risk of developing less common tumor types such as urothelial cancer (UC) (Järvinen *et al*., 2009). We have recently shown a significantly increased incidence of UC in the Danish Lynch syndrome population, which applies to both genders and increases with age (Therkildsen *et al*., 2017). The upper urinary tract urothelial carcinomas (UTUC) are particularly overrepresented in Lynch syndrome with cumulative risks estimated between 6.0 and 15.4% for UTUC and 2.4–9.0% for bladder UC (Engel *et al*., 2012; van der Post *et al*., 2010; Watson *et al*., 2008). Development of UC in Lynch syndrome is associated with mutations in the *MSH2* gene with such mutations in 65–70% of the UC in Lynch syndrome (Engel *et al*., 2012; Joost *et al*., 2015; van der Post *et al*., 2010; Skeldon *et al*., 2013).

Urothelial carcinomas of the upper urinary tract and the urinary bladder are morphologically and biologically similar (Krabbe *et al*., 2014). Differences in mutational profiles have been reported in high‐grade tumors with more frequent mutations in *FGFR3*,* HRAS*, and *CDKN2B* in UTUC and in *TP53* and *RB1* in bladder UC (Sanford *et al*., 2015; Sfakianos *et al*., 2015). In assumingly sporadic tumors, defective mismatch‐repair (MMR) is found in 11% of UTUC and in variable frequencies, most likely reflecting diverse assessment principles, in bladder UC (Catto *et al*., 2003; Metcalfe *et al*., 2018). Gene expression profiles have defined recurrent molecular subtypes in bladder UC and UTUC (Cancer Genome Atlas Research Network, 2014; Choi *et al*., 2014; Damrauer *et al*., 2014; Moss *et al*., 2017; Sjödahl *et al*., 2012), but have not been investigated in tumors from patients with Lynch syndrome. Here, we define molecular subtypes of UC linked to Lynch syndrome with comparison to a stage‐matched cohort of sporadic UC using both mRNA and immunohistochemically based expression signatures.

## 2 Materials and methods

### 2.1 Patient and tumor selection

Eligible patients with urothelial cancer (UC) were identified through the national Danish hereditary nonpolyposis colorectal cancer (HNPCC) registry as previously described (Joost *et al*., 2015). In brief, 92 UC had been surgically removed from 65 patients with Lynch syndrome between 1982 and 2013 of which tissues from 41 unique patients were available for analysis as no patient with samples from more than one tumor was available. All samples were investigated for microsatellite instability (MSI) with 19 tumors showing an MSI phenotype as previously described (Joost *et al*., 2015). The tumors developed in MMR mutation carriers (*n* = 40) or in deceased first‐degree relatives, in which gene carrier status could not be evaluated through tumor analyses (*n* = 1). Pathologic re‐evaluation was performed, tumor stage was determined according to the TNM 2009 classification, and tumor grading was applied using the WHO 1999 and 2004 systems. Surgery included transurethral resections of bladder cancers and nephroureterectomies for upper urinary tract tumors. The study was approved by the Scientific and Ethical Committee at the Capital Region of Denmark (HD‐2007‐0032 and H‐17001916) and the Danish Data Protection Agency (2007‐58‐0015 and AHH‐2017‐071).

### 2.2 RNA extraction and mRNA profiling

Total RNA was extracted from FFPE tissue blocks, amplified and labeled using the SensationPlus labeling kit, and hybridized to Gene ST 1.0 Affymetrix microarrays at the SCIBLU genomics facility in Lund, Sweden. Raw data are deposited at the Gene Expression Omnibus repository (http://www.ncbi.nlm.nih.gov/geo/query/acc.cgi?acc=GSE104922). RNA subtype classification of urothelial Lynch syndrome tumors was compared to a reference dataset of 307 advanced, subtype classified bladder tumors (http://www.ncbi.nlm.nih.gov/geo/query/acc.cgi?acc=GSE83586). Direct comparison to other large available datasets, such as TCGA, was not performed due to differences in data type (microarray versus RNA‐Seq, and sample quality FFPE‐RNA versus fresh‐frozen‐RNA). For details on mRNA profiling and subtype classification, Appendix S1.

### 2.3 Tissue microarrays (TMA) and immunohistochemistry (IHC)‐based subtype classification

For 36 of 41 tumors, sufficient tumor tissue to obtain two 1.0‐mm tissue cores was available to construct a TMA. Tumors were classified as urothelial‐like (Uro), basal/SCC‐like and genomically unstable (GU) based on a previously described IHC‐classifier (Pradere *et al*., 2017). For details Appendix S1.

### 2.4 Lynch syndrome in sporadic bladder cancer

To investigate undiagnosed Lynch syndrome in sporadic bladder cancer, we performed immunostaining for MLH1, MSH2, and MSH6 in an extended population‐based cohort (*n* = 383) (Sjödahl *et al*., 2017) using antibodies against MLH1 (ES05, dilution 1 : 50, Dako, Glostrup, Denmark), MSH2 (FE11, dilution 1 : 50, Dako), and MSH6 (EP49, dilution 1 : 50, Dako) and the Dako Envision™ FLEX+ detection kit (Dako) following manufacturer's instruction. In brief, 5 μm sections were deparaffinized and rehydrated and heat‐induced epitope retrieval was performed using a PT Link (Dako) for 20 min at pH 9. The antibody stainings were performed in a Dako Autostainer Plus (Dako) using the Envision™ FLEX+ Mouse or Rabbit LINKER (Dako). Sections were counter‐stained with hematoxylin and eosin, dehydrated, and preserved with coverslips using Permount™ mounting medium (Fisher Scientific, Hampton, NH, USA).

### 2.5 Statistical analysis

Overall survival (defined as time form surgery to date of death) for the 41 patients with Lynch syndrome urothelial cancer was compared to a reference cohort of sporadic bladder urothelial cancer (Sjödahl *et al*., 2012). To minimize the effect bias from T‐stage, identical proportions of Ta/T1/ ≥ pT2 were achieved by randomly excluding 53 pT1 and one ≥ pT2 tumor, leaving 114 pTa, 42 pT1, and 90 ≥ pT2 tumors in the external survival dataset. For Kaplan–Meier visualization of overall survival, data were censored at 150 months, which affected eight nonevents and one event, all in the Lynch syndrome subset. Differences in survival were evaluated using logrank test and univariate Cox proportional hazard ratio. Associations between clinical characteristics and molecular data were investigated using Fisher's exact test. Bonferroni corrections were used whenever relevant.

## 3 Results

### 3.1 Patient characteristics

The 41 Lynch syndrome‐associated UCs were located in the bladder (*n* = 19), the ureter (*n* = 14), and the renal pelvis (*n* = 8) (Table 1). IHC‐analysis showed loss of protein expression corresponding to the disease‐predisposing MMR gene mutation in 98% of the samples, of which 46% were microsatellite instable and 54% were microsatellite stable. The majority of tumors (71%) developed in *MSH2* mutation carriers. The tumors developed at mean age of 61 years (range 36 to 80 years) and with a preference for females (59%). Tumor stage was pTa or pT1 in 68% of the bladder cancers and in 59% of the upper urinary tract tumors. Previous metachronous cancer had developed in 32 patients and included 32 colorectal cancers, 12 endometrial cancers, and 18 UC.

**Table 1:** Descriptive data of the Lynch syndrome‐associated UC. Description of the clinical and pathological tumor characteristics in the 41 urothelial carcinomas in patients with Lynch syndrome‐associated UC. MSI = microsatellite instability and MSS = microsatellite stable tumors according to all five mononucleotide markers BAT‐25, BAT‐26, NR‐21, NR‐24, and MONO‐27 used (5). MSI‐low (MSI‐L) indicates instability for one marker and MSI‐high (MSI‐H) for ≥ two markers

Stage	Numbers (*n* = 41)	%
pTa	19	47
pT1	7	17
pT2	10	24
pT3	5	12
Grade (WHO 1999/2004)		
G1/LG	2	5
G2/HG	23	56
G3/HG	16	39
Gene affected		
MLH1	5	12
MSH2	29	71
MSH6	7	17
Carrier status		
First‐degree relative	1	2
Carrier	32	78
Obligate carrier	8	20
MSI status		
MSS	22	54
MSI‐L	7	17
MSI‐H	12	29
Anatomic site		
Bladder	19	46
Ureter	14	34
Renal pelvis	8	20

### 3.2 Characterization of Lynch syndrome‐associated tumors by gene expression profiling

**Figure 1:**
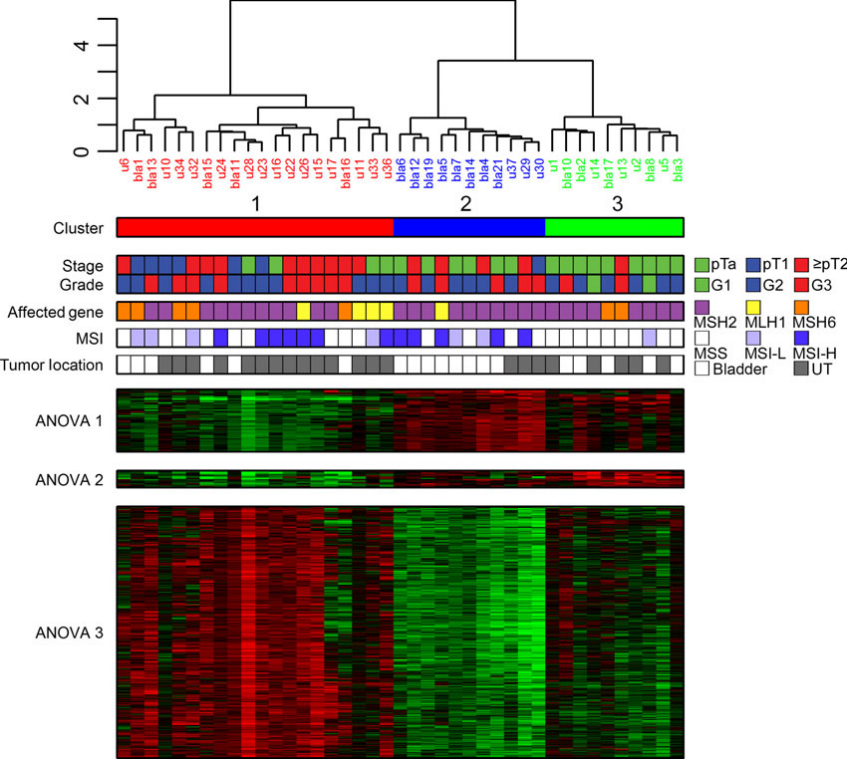
Gene expression analysis of Lynch syndrome‐associated UC. Gene expression data from 41 Lynch syndrome‐associated UC were median centered and subjected to hierarchical clustering analysis (Ward's algorithm, Pearson correlation). The three clusters identified were associated with tumor stage, grade (WHO 1999), and microsatellite instability, but not with affected gene. Heatmaps show three patterns of differentially expressed genes identified by three‐group ANOVA between Cluster 1 and Cluster 3. MSS = microsatellite stable; MSI‐low (MSI‐L) indicates instability for one marker and MSI‐high (MSI‐H) for ≥ two markers. UT = Upper urinary tract.

We performed unsupervised hierarchical clustering of the Lynch syndrome‐associated tumors (Fig. 1), using the top varying genes (*n* = 7569). This produced three major clusters, where Cluster 1 (*n* = 20) contained the majority of invasive tumors (> Ta) (4 pTa, 6 pT1, and 10 ≥ pT2). Cluster 2 (*n* = 11) contained 6 pTa, one pT1, and four ≥ pT2, and Cluster 3 (*n* = 10) contained 9 pTa and one ≥ pT2 tumor. The major division in the clustering was related to tumor stage, which showed significant difference between clusters (pTa versus ≥ pT1, *P* < 0.001). Furthermore, 65% of the UTUC were located in Cluster 1 and 72% of bladder tumors in Cluster 2 (Fisher's exact test, *P* = 0.03). None of the clusters were specifically associated with mutations in *MSH6*,* MLH1*, or *MSH2*, but there was a significant difference in the rate of microsatellite instability (MSI) among the clusters, with Cluster 3 showing the lowest proportion of MSI (Fisher's exact test, *P* = 0.01). A three‐group ANOVA test indicated 806 significant genes (*P* < 0.01, Bonferroni corrected) with the majority showing upregulation in Cluster 1. Gene ontology analysis showed broad enrichment of terms related to, for example, cell cycle regulation, metabolic and catabolic processes, and RNA processing (Table S1). Comparing global mRNA expression between Lynch syndrome‐associated bladder tumors and upper urinary tract UC revealed no significant differences in gene expression patterns using *t*‐test and SAM analysis (data not shown).

### 3.3 Lynch syndrome‐associated UC in relation to sporadic bladder cancers

**Figure 2:**
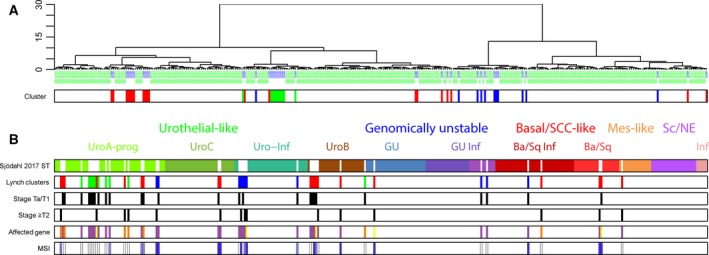
Lynch syndrome‐associated UC co‐segregates with molecular subtypes of sporadic bladder cancer. (A) The 41 Lynch syndrome‐associated UCs were recentered with a cohort of advanced sporadic UC and subjected to hierarchical clustering analysis (Ward's algorithm, Pearson correlation). The results indicate that some Lynch syndrome tumors cocluster, but Lynch syndrome UCs are found in all branches of the cluster dendrogram, indicating that Lynch tumors are not fundamentally different from sporadic UC. (B) Lynch syndrome‐associated UC cases were mapped to bladder cancer molecular subtypes by placing each Lynch syndrome sample next to the sporadic UC sample with highest correlation (Pearson‐r) in the subtype‐ordered combined dataset. (Mes‐like = mesenchymal‐like; Sc/NE = small‐cell/neuroendocrine‐like; GU = genomically unstable; Ba/Sq = basal/squamous‐like; Inf = infiltrated).

To compare Lynch syndrome‐associated and sporadic UC, we combined mRNA data from the 41 Lynch syndrome tumors with data from 307 sporadic advanced bladder cancers (Sjödahl *et al*., 2017). Unsupervised hierarchical cluster analysis showed that Lynch syndrome tumors were distributed among the sporadic bladder cancers and did not form a distinct group separated from the main cohort of sporadic tumors. The Lynch syndrome tumors formed two aggregations consisting of Cluster 1 tumors and Cluster 3 tumors, respectively (Fig. 2A). Hence, the expression profiles of Lynch syndrome tumors seem to be more similar to each other than to sporadic tumors (Fig. 2A). To examine how the Lynch syndrome samples related to the molecular subtypes of bladder cancer, we arranged the reference dataset according to molecular subtype (Sjödahl *et al*., 2017) and inserted the Lynch syndrome UC into the dataset by placing them adjacent to the most similar sporadic tumor (Fig. 2B). The majority (33/41) grouped with bladder cancer of the Uro subtypes (18 UroA‐Prog, two UroC, six Uro‐Inf, and seven UroB) (Fig. 2B). The progressed UroA group (UroA‐Prog) contained the majority of lower stage tumors in the reference dataset, and accordingly, 9 of 10 low‐stage Cluster 3 Lynch syndrome tumors grouped with this subtype. Among the remaining eight Lynch syndrome samples, three grouped with the genomically unstable (GU), four with the basal/SCC‐like, and one with mesenchymal‐like bladder tumors. No significant difference in stage (pTa/pT1 versus ≥ pT2) or affected MMR gene of Lynch syndrome tumors was observed when grouped by subtype. Similarly, we found no significant difference in the rate of MSI between cases clustering with the UroA‐Prog (MSI = 6, MSS = 11) versus those clustering with the other Uro subtypes (MSI = 6, MSS = 4, *P* = 0.3), nor when comparing the UroA‐Prog group to all other Lynch syndrome cases (MSI = 13, MSS = 10, *P* = 0.2).

### 3.4 Molecular pathological analysis of Lynch syndrome‐associated urothelial carcinomas

**Figure 3:**
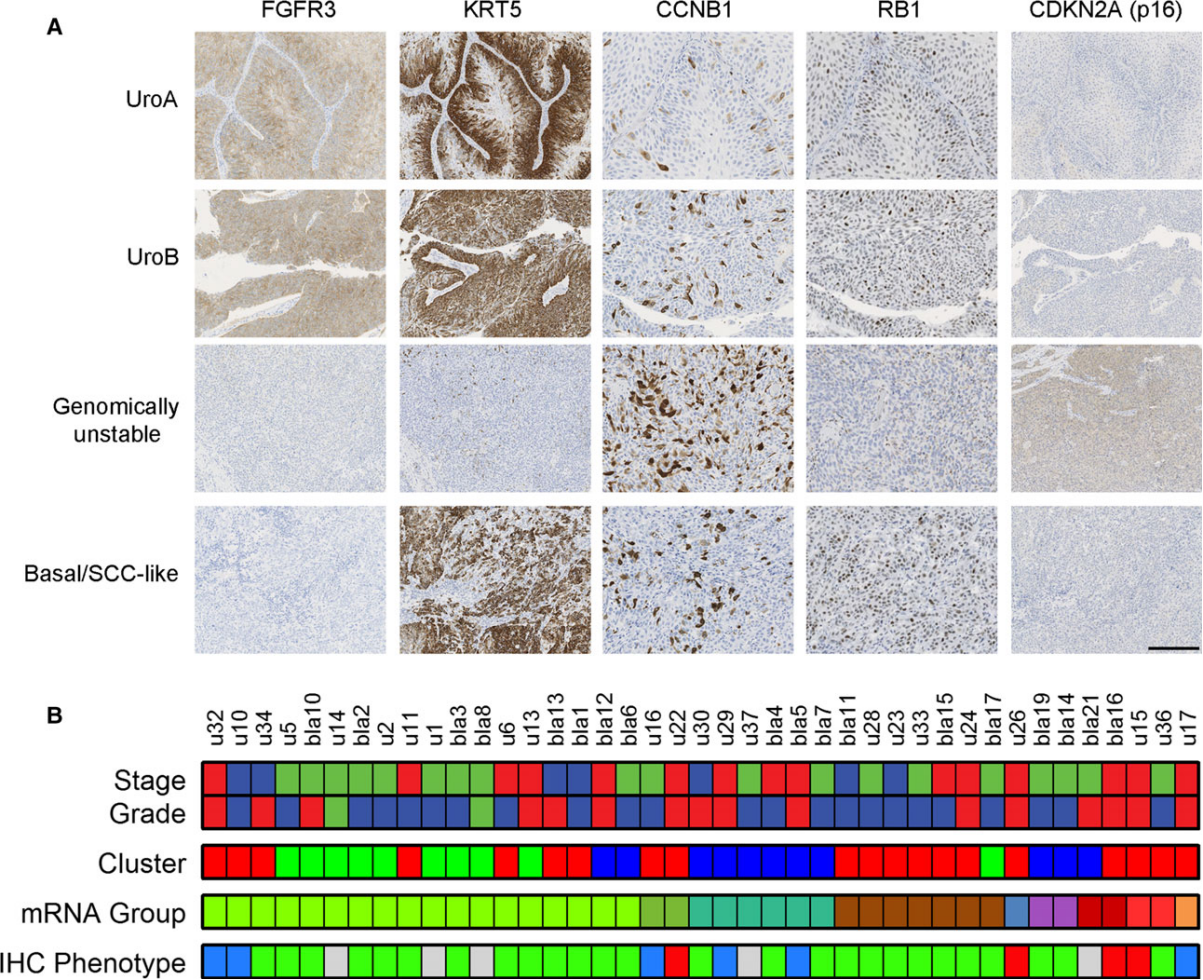
Molecular pathological analysis of Lynch syndrome‐associated UC shows strong similarity to sporadic UC phenotypes. (A) Four examples of 37 analyzed Lynch syndrome‐associated UC showing IHC marker profiles typical for the bladder cancer subtypes UroA, UroB, genomically unstable, and basal/SCC‐like. Scale bar, 200 μm. B) IHC‐based subtype classification was based on IHC‐scores, tumor grade (WHO1999), and urothelial‐like histology. IHC‐subtype is shown in relation to tumor stage, tumor grade (WHO1999), Lynch syndrome cluster (color‐coded as in Figure 1), and mRNA subtype (color‐coded as in Figure 2B). IHC‐phenotype were color‐coded as follows; green = urothelial‐like (Uro); blue = genomically unstable (GU); red = basal/SCC‐like; gray = missing data.

The TMA sections were stained for markers associated with molecular subtype, that is, antibodies for FGFR3, CCNB1, KRT5, RB1, and CDKN2A (p16), and examined for maintained urothelial‐like stratification of cell layers. The results were strongly reminiscent of sporadic bladder cancer, and 21 of 37 cases showed urothelial‐like histology, low stage, and grade, thus sharing characteristics with the Uro subtype. This was supported molecularly by stratified expression of basal urothelial markers; for example, KRT5 expression and proliferation limited to the basal and suprabasal layers, and high tumor‐cell expression of FGFR3 in all cell layers (Fig. 3A). Among the non‐Uro classified cases, six were classified as GU with high levels of CDKN2A (p16) and CCNB1, while four were classified as basal/SCC‐like due to up‐regulation of KRT5 and downregulation of FGFR3 (Fig. 3B). The IHC staining did not differ in patterns compared to sporadic UC. For example, the GU IHC‐class contained cases with RB1 loss and showed higher CCNB1 staining than Uro‐classified cases (group mean 0.27 versus 0.13, *t*‐test, *P* = 1.8 e‐5) and four of six were of stage ≥ pT2 (Fig. 3B). The basal/SCC‐like cases were all of stage ≥ pT2 and showed nonstratified KRT5 expression (Fig. 3A,B). The IHC‐class corresponded to the subtype defined by mRNA expression (Fig. 2B) in the sporadic UC dataset in 69% of the cases (Fig. 3B). Taken together, these data indicate that UCs in patients with Lynch syndrome molecularly are comparable to sporadic urinary UC.

### 3.5 Overall survival for Lynch syndrome‐associated and sporadic urothelial cancer

**Figure 4:**
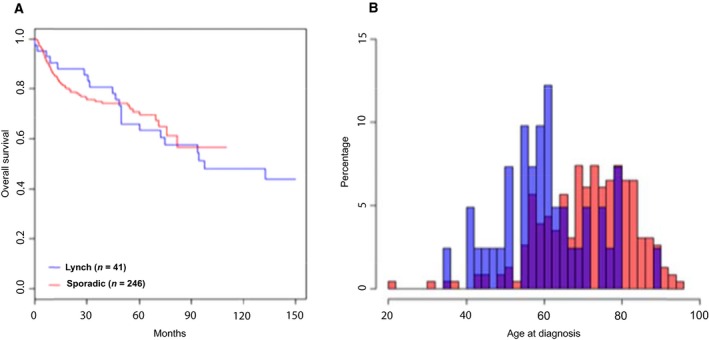
Patients with Lynch syndrome‐associated UC have similar overall survival, but are diagnosed at younger age than patients with sporadic UC. (A) Kaplan–Meier estimates of overall survival for the 41 Lynch syndrome cases compared to a stage‐matched subset of a cohort of sporadic UC of the bladder. No significant differences were observed (*P* = 0.81). (B) Histogram showing patients age at first UC diagnosis for all 41 Lynch syndrome cases and all cases in the sporadic cohort for which this information was available (*n* = 230). *Y*‐axis is recalculated to percentages in order to account for large differences in cohort size.

**Figure 5:**
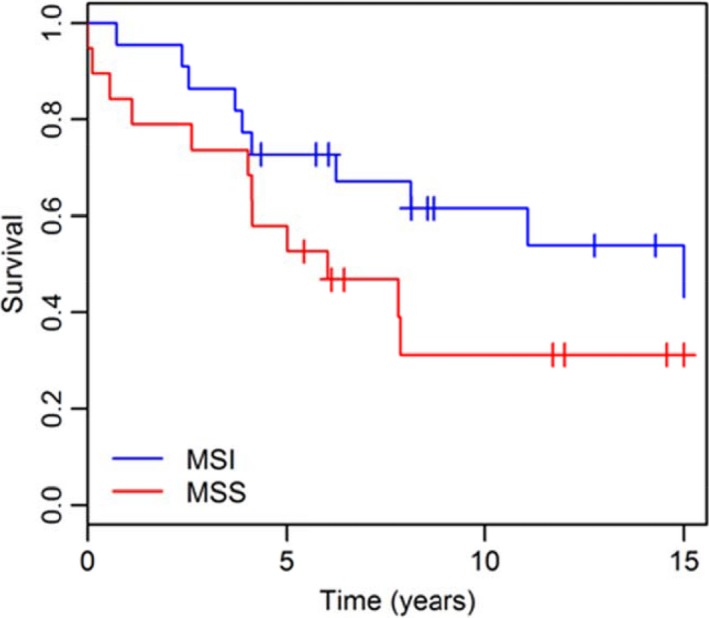
Overall survival stratified by MSI/MSS. Overall survival for MSI (blue) and MSS (red) Lynch syndrome‐associated tumors (*P* = 0.14).

The 10‐year crude survival for Lynch syndrome patients with UC was 50%, which was similar for stage‐matched sporadic bladder cancer cohort (*n* = 246) (Sjödahl *et al*., 2012). Univariate analysis indicated no difference in outcome between the groups (HR: 1.07 95% CI: 0.63–1.82, logrank‐*P* = 0.81) (Fig. 4A). Furthermore, fundamental patient characteristics such as gender distribution (Lynch syndrome: 24 females and 17 males; sporadic cancer: 80 females and 228 males, Fisher's exact test, *P* = 4.6 e‐5) and age at diagnosis (60 years for Lynch syndrome and 73.5 years for sporadic cancer; Mann–Whitney, *P* = 4.0 e‐7) (Fig. 4B) differed significantly between the two cohorts. Overall survival was not significantly influenced by MSI status within the MMR‐deficient Lynch syndrome cohort (Fig. 5).

### 3.6 Lynch syndrome in sporadic advanced bladder cancer

To investigate whether advanced sporadic bladder tumors include potentially undiagnosed Lynch syndrome patients, we performed IHC for MLH1, MSH2, and MSH6 in a population‐based cohort (*n* = 383) (Sjödahl *et al*., 2017). Only two tumors (0.5%) showed tumor‐specific loss of staining for MLH1 and MSH6, respectively, suggestive of Lynch syndrome and thus have a negligible impact on the results.

## 4 Discussion

Based on mRNA and IHC profiling, we demonstrate that Lynch syndrome‐associated UC segregates into three clusters associated with tumor stage, grade, and MSI status, which represent the main determinants of the global gene expression pattern (Fig. 1). When analyzed in the context of sporadic UC, Lynch syndrome‐associated tumors clustered within the established molecular subtypes and together with sporadic tumors. Both mRNA‐based and IHC‐based molecular classifications resulted in the expected frequencies of molecular subtypes given the stage distribution in the cohort, which suggests that the predefined molecular subtypes dominate over genetic predisposition. At the molecular pathology level, most tumors demonstrated an urothelial‐like (Uro) phenotype with low stage/grade, retained stratification of basal‐intermediate cell layers, and frequent FGFR3 expression. Among T1 and ≥ T2 cases, some tumors showed typical basal/squamous‐like or genomically unstable phenotypes, confirming that all the three major UC phenotypes are represented also in Lynch syndrome. Data that compare molecular subtypes in UC of the upper and the lower urinary tract are scarce. In a gene expression study of 12 UTUC and 20 bladder UC, the basal subtype was suggested to be less frequent in UTUC (Sanford *et al*., 2015). Mutations in UTUC and bladder UC differed only for *HRAS, CDKN2B, TP53, RB1,* and *ARID1A* when investigated in a set of 59 high‐grade UTUC and 102 high‐grade bladder UC (Sanford *et al*., 2015; Sfakianos *et al*., 2015). Gene expression patterns did not differ between the 19 bladder UC and 22 UTUC in the present study; however, limited sample size and heterogeneous stage distribution preclude any firm conclusions.

The comparison with sporadic UTUC and bladder cancer revealed a female preponderance and younger age at first UC diagnosis for patients with Lynch syndrome. As previously reported (Pradere *et al*., 2017), UC linked to Lynch syndrome develops at a lower age than sporadic UC with mean 13.5 years earlier age at onset in our study. Lynch syndrome‐associated tumor may have a better response to adjuvant chemotherapy after radical nephroureterectomy (Hollande *et al*., 2014). Long‐term outcome is, however, also influenced by the high frequency of metachronous tumor development, which affected 78% of the Lynch syndrome patients in our study with metachronous development of 32 colorectal cancers and 18 UC.

Increased awareness of Lynch syndrome among patients with UC, particularly tumors of the upper urinary tract, is warranted as Lynch syndrome remains underdiagnosed (Audenet *et al*., 2012; Catto *et al*., 2003). Failure to recognize Lynch syndrome‐associated cases implies missed chances for genetic counseling, surveillance programs for family members at increased risk, and potential precision therapy with anti‐PD‐1 inhibition for MMR‐deficient UC (Le *et al*., 2015). Our findings suggesting that the predefined molecular subtypes dominate over genetic predisposition also emphasize the need to screen UC, or at least UTUC for MMR‐defective tumors to identify patients with Lynch syndrome. Identified individuals diagnosed with UC should probably be subjected to lifelong surveillance, even if oncogenetic counseling based on gene mutation and gender might modify the risk of a recurrent tumor (Therkildsen *et al*., 2017). How gene‐mutation carriers, especially MSH2, should be surveyed for UC is currently not known, and different methods are recommended; however, urinary cytology has a low sensitivity (29%) and thus not sufficient (Myrhøj *et al*., 2008).

Colorectal cancers that develop in Lynch syndrome show a better disease‐specific survival than sporadic colorectal cancer, most likely due to the immunogenic signatures in MMR‐defective tumors (Boland, 2016; Maccaroni *et al*., 2015). This hypothesis is further supported by the finding that MMR status predicts clinical benefit from PD‐1 blockade in colorectal cancer (Le *et al*., 2015). The availability of immunotherapy for patients with MMR‐defective tumors offers new hope in Lynch syndrome. Checkpoint inhibitors are since 2016 also available in UC following the approval of the PD‐L1 inhibitor Atezolizumab in advanced UC (Rosenberg *et al*., 2016).

This report represents the first molecular subtyping of UC in Lynch syndrome and despite limited in size represents a large collection of this rare tumor entity with disease‐predisposing MMR gene mutations/MMR protein loss in concordance with the germline mutation proven in 98% of the tumors. The MSI frequency of 46% is comparable to other studies, suggestive of a reduced sensitivity for this method in UC with the currently available markers (Amira *et al*., 2003). Study limitations relate to lack of data on disease‐specific survival and limited availability of publically available data on gene expression signatures from sporadic UTUC (Moss *et al*., 2017).

## 5 Conclusions

Urothelial carcinoma from patients with Lynch syndrome cluster according to MSI status, tumor location, and tumor grade and within the molecular subtypes established in sporadic UC. Urothelial‐like tumors predominate, which is consistent with the pathological stage distribution. These data support that application of novel molecular diagnostics and targeted therapeutics developed in sporadic UC may be relevant also for patients with Lynch syndrome.

## Author contributions

CT and MF had the idea for this study. MJ, GS, and PE undertook the tissue preparations and analyses; MH, CT, and FL supervised the acquisition of the data. CT, PE, and GS undertook the statistical analysis; all authors contributed to interpretation of the results and wrote the article, and contributed to the content. All authors approved the final version of the manuscript, including the authorship list.

## Supporting information


**Table S1.** Differentially regulated genes and gene ontology terms in clusters 1–3.

**Appendix S1.** mRNA profiling, molecular subtype classification and TMA and IHC‐based subtype classification.
