# Supplementary material for: Molecular subtype classification of urothelial carcinoma in Lynch syndrome
Source: Mol Oncol. 2018 Jun 19;12(8):1286–95. doi: 10.1002/1878-0261.12325 (PMC6068353; doi:10.1002/1878-0261.12325)
Supplement: Supplementary file 2 — Appendix S1. mRNA profiling, molecular subtype classification and TMA and IHC‐based subtype classification. [file MOL2-12-1286-s002.docx]

**Appendix S1**

**RNA extraction and mRNA profiling**

Formalin-fixed paraffin-embedded tissue blocks were sectioned and stained with Hematoxylin and Eosin (H&E). Tumor containing areas were macro-dissected by marking tumor-containing area on the H&E slide. The marked area was scratched into the tissue block, and 4-10 10-µm sections were taken. FFPE tissue flakes containing the marked areas were deparaffinized and rehydrated by serial incubation with pure xylene, 2:1 xylene/ethanol, and pure ethanol. The dried tissue pellet was treated with proteinase K in lysis buffer (HighPure, Roche, Basel, Switzerland) overnight and the resulting lysate was passed through RNA isolation columns (HighPure, Roche) with on-filter DNAse I treatment according to the manufacturer’s instructions. Total RNA yield was measured using Nanodrop and stored at -80 °C. Samples with a total RNA yield above 0.75 µg (n=41) were selected for mRNA profiling. RNA samples were amplified and labeled using the SensationPlus kit (Affymetrix, Santa Clara, CA, USA). Labeling of RNA and hybridization to single chip Gene ST 1.0 Affymetrix microarrays was performed at the SCIBLU genomics facility in Lund, Sweden. Raw gene expression data for the 41 cases is available in the Gene Expression Omnibus (GEO) depository under the identifier (GSE104922).

**Gene expression data analysis and molecular subtype classification**

As a reference for mRNA based subtype classification we used a cohort of 307 advanced bladder tumors, in which molecular subtype has been established by mRNA expression profiling (GSE83586) and immunohistochemistry (Sjödahl *et al*., 2017). Both data sets were generated using the same extraction and hybridization methodology at the same facility. In order to classify the Lynch syndrome urothelial tumors into bladder cancer molecular subtypes, we constructed a combined dataset by merging raw data from the two cohorts and performing all pre-processing steps: RNA normalization, quantile-normalization, intensity filtering of probes, followed by probe annotation and filtering, and finally batch correction using COMBAT (Johnson *et al*., 2007). The step-by-step pipeline for data processing is shown below. For analyses of Lynch syndrome-associated urothelial tumors alone, the data set was median-centered on the Lynch syndrome cases only. This centering was used for clustering (C1-C2-C3, as shown in Figure 1). Three-group ANOVA test was used to identify significantly differentially expressed genes between the Lynch syndrome samples. Hierarchical clustering was performed in R using Ward’s algorithm and Pearson-R as distance measure. Gene ontology enrichments were calculated using the Panther Version 12 (pantherdb.org). For molecular subtype analysis in the context of the sporadic bladder cancer cohort, the Lynch syndrome urothelial tumor data was median-centered in the combined set of 348 tumors. For each Lynch syndrome urothelial tumor we then identified the nearest neighbor (pearson-R) in the reference cohort, and in this way placed the Lynch urothelial tumor into the context of advanced bladder cancer molecular subtypes.

Pipeline for gene expression analysis:

1. Raw data

2. RMA normalization

3. Quantile Normalization

4. Intensity filtering, removing all probe sets below detection level in 80% or more of the samples

5. Probe set annotation to gene symbol, removal of control probes, and merging of probe set by gene symbol

6. Application of COMBAT to adjust data for minor differences between labeling batches.

7. Median centering

7.1 Urothelial Lynch syndrome cases alone (for separate analyses)

7.2 Urothelial Lynch syndrome cases with (GSE83586) reference urothelial carcinoma data set, for assigning molecular subtype by nearest neighbor.

**Tissue microarrays (TMA) and immunohistochemistry (IHC)-based subtype classification**

Placement of tissue cores was independent of prior macrodissection for RNA extraction. Primary antibodies against FGFR3 (#4574, Cell Signaling, Danvers, Massachusetts, USA), KRT5 (RM-2106, LabVision, Fremont, CA, USA), CCNB1 (1495-1, Epitomics, San Francisco, CA, USA), RB1 (#9309, Cell Signaling), and CDKN2A (p16) (550834, BD Biosciences, San Jose, CA, USA) were used as described previously (Sjödahl *et al*., 2013). Slides were scanned (AxioScan Z1, Zeiss, Oberkochen, Germany) and evaluated using the digital pathology platform PathXL (PathXL Ltd, Belfast, UK). Evaluation of IHC was performed on an intensity scale (0−3, used for FGFR3 and p16), a percentage scale for positive tumor cells (0−9, used for CCNB1 and RB1), or both multiplied (0−2.7 used for KRT5). Two observers (GS and CT) performed IHC evaluation with good concordance (Spearman correlation ranging from 0.77−0.96 for the five markers). The mean value from the two observers was used.
